# Phenotyping the Spectrum of Traumatic Brain Injury: A Review and Pathway to Standardization

**DOI:** 10.1089/neu.2021.0059

**Published:** 2021-11-23

**Authors:** Mary Jo Pugh, Eamonn Kennedy, Eric M. Prager, Jeffrey Humpherys, Kristen Dams-O'Connor, Dallas Hack, Mary Katherine McCafferty, Jessica Wolfe, Kristine Yaffe, Michael McCrea, Adam R. Ferguson, Lee Lancashire, Jamshid Ghajar, Angela Lumba-Brown

**Affiliations:** ^1^ Informatics, Decision-Enhancement and Analytic Sciences Center, VA Salt Lake City, Salt Lake City, Utah, USA.; ^2^ Department of Internal Medicine, Division of Epidemiology, University of Utah School of Medicine, Salt Lake City, Utah, USA.; ^3^ Cohen Veterans Bioscience, New York, New York, USA.; ^4^ Department of Rehabilitation and Human Performance, Department of Neurology, Icahn School of Medicine at Mount Sinai, New York, New York, USA.; ^5^ Department of Neurology, University of California San Francisco, California, USA.; ^6^ San Francisco Veterans Affairs Medical Center, San Francisco, California, USA.; ^7^ Department of Psychiatry, University of California San Francisco, California, USA.; ^8^ Department of Neurosurgery, Medical College of Wisconsin, Milwaukee Wisconsin, USA.; ^9^ Department of Neurological Surgery, University of California San Francisco, California, USA.; ^10^ San Francisco Veterans Affairs Health System, San Francisco, California, USA.; ^11^ Department of Neurosurgery, Stanford University School of Medicine, Stanford, California, USA.; ^12^ Department of Emergency Medicine, Stanford University School of Medicine, Stanford, California, USA.; ^13^ Brain Performance Center, Stanford University School of Medicine, Stanford, California, USA.

**Keywords:** clinical profiles, clustering, coma, concussion, meta-analysis, phenotypes, subclassification, subtypes, traumatic brain injury

## Abstract

It is widely appreciated that the spectrum of traumatic brain injury (TBI), mild through severe, contains distinct clinical presentations, variably referred to as subtypes, phenotypes, and/or clinical profiles. As part of the Brain Trauma Blueprint TBI State of the Science, we review the current literature on TBI phenotyping with an emphasis on unsupervised methodological approaches, and describe five phenotypes that appear similar across reports. However, we also find the literature contains divergent analysis strategies, inclusion criteria, findings, and use of terms. Further, whereas some studies delineate phenotypes within a specific severity of TBI, others derive phenotypes across the full spectrum of severity. Together, these facts confound direct synthesis of the findings. To overcome this, we introduce PhenoBench, a freely available code repository for the standardization and evaluation of raw phenotyping data. With this review and toolset, we provide a pathway toward robust, data-driven phenotypes that can capture the heterogeneity of TBI, enabling reproducible insights and targeted care.

## Introduction

**FIG. 1.**
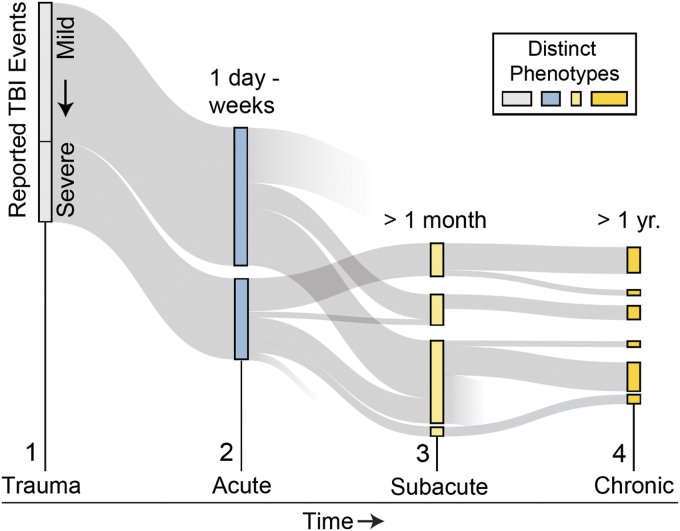
Conceptual overview of the progression and divergence of traumatic brain injury (TBI) phenotypes. TBI phenotypes (rectangles) emerge statistically (1–4) from the presence and duration of symptoms/impairments. The prevalence (rectangle size) of phenotypes can vary and evolve over time and can signal recovery or decline. Color image is available online.

Traumatic brain injury (TBI) represents a spectrum of micro- and macroscopic brain injury, traditionally classified as mild, moderate, or severe.^1^ However, scientific literature supports that the spectrum of TBI also contains distinct clinical presentations or phenotypes that are dependent upon pre-morbid, post-morbid, and injury-related factors.^2–5^ TBI phenotypes include age, sex, genetics, baseline health, injury-related factors (e.g., severity of injury and coexisting injuries), past medical history, and comorbidities. Over time, persisting symptoms may give rise to adverse outcomes^6,7^ and divergent recovery pathways (Fig. 1). Cumulatively, this heterogeneity creates challenges for efficiently strategizing TBI care and predicting recovery.

To address the need for improved prognostication, researchers have applied a range of approaches for identifying TBI phenotypes (Table 1). We review these approaches, aggregate trends, and describe consistencies across reports, but also note diverging methodologies, varying descriptions of phenotypes, inconsistent use of terms, and a general lack of shared standards. Further, whereas many studies include the full spectrum of TBI severity in a single analysis (i.e., identifying traits/outcome similarities regardless of severity), others explore phenotypes within just one severity window (e.g., mild TBI only). Although we note differences in measure collection, inclusion criteria, and analytical framework throughout the literature, addressing shortcomings in TBI phenotyping offers opportunities to enhance precision health, enable targeted care, and improve patient outcomes.

**Table 1.** TBI Phenotypes

Study	Phenotype domain	Dim. reduction	Age	Population	Injury stage at assessment	Severity	Measures	Subgroups or clusters	Approach
1. DeJong and Donders, 2010	Cognitive	No	16–79	Civilian	Chronic	All severities	CVLT-II	Six subgroups: four replicated in mTBI; five replicated in severe TBI	Clustering (fastclus); Agglomerative clustering on variance; *k*-means labeling
2. Mottram and Donders, 2006	Cognitive	No	6–16	Civilian	Chronic	All	CVLT-C	Four clusters based on level and pattern of performance	Clustering (fastclus); Complete linkage procedure (reliability check)
3. Sherer et al., 2017	Cognitive	No	16–70	Civilian	Chronic	All	TBI-QOL, NSI, EQOL, FADGF, PART-O	Five clusters that had significantly different results on PART-O scale	Clustering; agglomerative clustering on variance; *k*-means labeling
4. Pugh et al., 2019	Comorbid factors	No	18–65	Military/Veterans	Chronic	Mild	GCS, diagnosed health conditions	Five comorbidity trajectories: moderately healthy stable; moderately healthy decline; mental health, polytrauma stable; polytrauma with improvement	LCA, logistic regression
5. Lumba-Brown et al., 2020	Post-concussive symptom focused	No	6+	All populations	Acute	Mild	Concussion Symptom Scales and other indicators	Five concussion subtypes: cognitive, ocular-motor, headache/migraine, vestibular, anxiety/mood (sleep disturbance also associated)	Literature review and meta-analysis
6. Maruta et al., 2018	Symptom	No	12–30	Civilian athletes	Acute	Mild	RPQ	Six classes of symptoms: cognitive/fatigue, vestibular, oculomotor, anxiety/mood, migraine, cervical/sleep	Binomial tests; expert assignment; subtype prevalence overlap analysis
7. Velikonja et al., 2010	Emotional and behavioral	No	15–69	Canadian clinical cohort	Chronic	All	PAI	Seven clusters: multiple symptoms, somatic/depressive symptoms, normal, depression, substance use/antisocial, normal (minimizing), multiple symptoms/bipolar	Split sample; three clustering methods; hierarchical, agglomerative, *k*-means, linkage, iteration
8. Warriner et al., 2003	Emotional and behavioral	No	15–69	Canadian clinical cohort	Chronic	Mild-moderate (75% of patients)	MMPI	Six injury outcome subtypes: normal function, mild somatic/pain concerns, disinhibition/externalizing behavior, internalizing behavior, externalizing and somatic behavior	Split sample; three clustering methods; hierarchical, agglomerative, *k*-means, linkage, iteration
9. Juengst et al., 2017b	Emotional, cognitive, and behavioral	No	16–70	Civilian	Chronic	All	PHQ-9; PANAS; NTB; FSBS	Temporal evolution of emotional, cognitive and behavioral clusters; <6-month injury: clustered along continuum of emotion/behavior symptoms; >6-month injury: complex symptom patterns	Cross-lagged panel analysis, structural equation modeling
10. Nielson et al., 2017	Outcome, biomarkers	Yes	43.3 ± 18.5	Civilian	Chronic	All	Injury character, neuroimaging, PTSD Checklist; WAIS; CVLT	Two broad topological node groups, six nodal extrema. One mTBI node reflecting unfavorable outcomes on GOSE 3–6 months, (including PTSD) associated with PARP1, ANKK1, COMT, and DRD2	Topological data analysis with third-party software, linear models
11. Goldsworthy and Donders, 2019	Personality	No	18–75	Civilian	Chronic	All	MMPI-2-RF	Four clusters: clusters 1 and 4 differed by profile elevations; clusters 2 and 3 varied in pattern. Pre-morbid factors separated clusters.	Clustering (fastclus)
12. Kennedy et al., 2015	Personality	No	19–49	Military	Chronic	Mild	PAI	Four clusters: high distress, moderate distress, somatic distress, no distress	Clustering, hierarchical, and *k*-means
13. Hellstrom et al., 2013	Symptom	No	16–55	Civilian	Chronic	Mild	RPQ	Four clusters: low symptoms, high symptoms, cognitive, somatic	Clustering, hierarchical, and *k*-means
14. Polimanti et al., 2017	Symptoms	No	18–46	Military post-9/11	Chronic	Unknown	GWAS; PCS	No significant association of post-concussive symptoms (PCS) with any genetic components; high infant HC-PRS was correlated with better recovery from concussion.	Genome-wide cross-phenotype analysis with PRSice, linkage disequilibrium, enrichment, regression
15. Stein et al., 2016	Symptom	No	18–46	Military post-9/11	Chronic	Mild	PCS	Severity of PCS associated with five traits: history of TBI, stress, more severe deployment-related events, LOC lapse of memory vs. LO attention	Zero-inflated negative binomial regression
16. Ensign et al., 2012	Psychosocial	No	6–20	Civilian	Chronic	Mild-severe	BASC-2	Six: two primary: Normal, Pervasive emotional difficulties; four less reliable: Mild Externalizing with 1) Depression, 2) Attention Problems, 3) Mild Depression, and 4) Mild Anxiety	Agglomerative hierarchical cluster analysis and simple UPGMA, Ward's methods
17. Hayman-Abello et al., 2003	Psychosocial	No	12–18	Civilian	Chronic	Mild-severe	CBCL	Four groups: Normal, Attention, Delinquent, and Withdrawn-Somatic	Q-factor analysis
18. Folweiler et al., 2020	Severity	Yes	18–70	Civilian	Acute	Mild-severe	GCS	Three patient phenotypes, two replicated across studies	GLRM; Gower's dissimilarity matrix K-nearest neighbor
19. Gravesteijn et al., 2020	Severity	Yes	50^a^ [30, 66]	Civilian	Acute	Mild-severe	Injury Mechanism Extracranial Injury GCS	Four clusters of severity associated with differential long-term outcomes	Bootstrap resampling with replacement; PCA and Gower's distance
20. Masino et al., 2018	Severity	Yes	18–70	Civilian	Chronic	Mild-severe	Baseline <24 h, CT scan, other intake history	Four distinct patient phenotypes that were associated with 90-day outcomes on 12 assessments	GLRM feature selection, Gower's distance, dissimilarity matrix
21. Si et al., 2018	Severity	No	16+	Civilian	Acute	Mild	GCS, clinical variables, GOSE, WAIS	Five mTBI subgroups: general, cognitive, functional, emotional, and somatic	Sparse hierarchical clustering with automated feature rejection/selection
22. Kucukboyaci et al., 2018	Comorbid and vulnerability	No	16–70	Civilian	Unspecified	Not specified	Demographics, psychosocial	Four clusters: 1) high substance use and psychiatric history; 2) race/ethnic minority, limited English proficiency; 3) minority with substance use, incarceration, and homelessness; and 4) elderly with complex comorbidity	Two-step clustering with log-linear differences and *k*-means
23. Yeates et al. 2019	General post-concussion	No	8–18 (8–12, 13–18)	Civilian	Acute - chronic	Mild	Pre-morbid, clinical. ACE, PCSI, SAC, and BESS	Four clusters found using pre-morbid history. Four clusters found using clinical data. Assessment at 4 and 12 weeks. Age, female sex, (anxiety), phenotypes increase PPCS risk.	LCA
24. Bailie et al. 2016	Psychosocial, cognitive, behavioral	No	18–56	Military	Chronic	Mild	Neurobehavioral Symptom Inventory and PTSD Checklist-Civilian Version (PCL-C)	Four subtypes: primarily psychiatric (post-traumatic stress disorder) group, a cognitive group, a mixed symptom group, and a good recovery group	Two-step clustering procedure (hierarchical clustering and *k*-means labeling) using average linkage/Pearson correlation as the proximity measure
25. Zimmermann et al. 2015	Cognitive, executive function	No	18+	Civilian	Chronic	All severities	Multiple Executive Function tasks	Three clusters: 1) inhibition, flexibility, and focused attention; 2) inhibition, flexibility, working memory, and focused attention; and 3) no expressive executive deficits	Hierarchical cluster analysis, tasks Z-scores, ANOVA
26. Howell et al. 2019	Post-concussive symptom focused	No	7–30	Civilian	Acute-chronic	Mild	Post-Concussion Symptom Scale	Five symptom domains: 1) somatic, 2) emotional, 3) sleep, 4) cognitive, and 5) vestibular-ocular	Linear regression model
27. Kontos et al. 2019	Post-concussive symptom focused	No	11–40	Civilian	Unspecified	Mild	Medical history, injury, clinical interview/examination notes, cognitive/vestibular/ocular tests	Six profiles: 1) cognitive/fatigue, 2) vestibular, 3) ocular, 4) post-traumatic migraine, 5) anxiety/mood, and 6) cervical	Blinded chart reviews by six clinicians determining the primary and secondary clinical profiles
28. Feddermann-Demont et al. 2017	Post-concussive symptom focused	No	Teens-adults (mean age 17)	Athletes	Unspecified	Mild	Symptom scales, neurocognitive tests, balance	Five domains: cognition, dizziness and balance, emotions, headache, and vision	Clinical comparison of post-concussive symptoms; subanalysis of predominant symptoms

^a^ Interquartile range.

AUDIT, Alcohol Use Disorders Identification Test; BDI-II, Beck Depression Inventory II; BASC-2, Behavior Assessment System for Children, Second Edition; UPGMA, between-group linkage; CVLT-C, California Verbal Learning Test–Children's Version; CVLT-II, California Verbal Learning Test–Second Edition; CBCL, Child Behavior Checklist; EQOL, Economic Quality of Life Scale; ESL, English as Second Language; FADGF, Family Assessment Device General Functioning Scale; FSBS, Frontal Systems Behavior Scale; GLM, general linear model; GLRM, generalized low-rank models; GWAS, genome-wide association study; GCS, Glasgow Coma Scale; GOSE, Glasgow Outcome Score-Extended; IHC, infant head circumference; LCA, latent class analysis; LOOCV, leave one out sample cross-validation; MMPI-2-RF, Minnesota Multiphasic Personality Inventory–2–Restructured Form; NSI, Neurobehavioral Symptom Inventory;,NTB, Neuropsychological Test Battery; PART-O, Participation Assessment with Recombined Tools-Objective; PHQ-9, Patient Health Questionnaire-9; PAI, Personality Assessment Inventory; PRS, Polygenic Risk Score; PANAS, Positive and Negative Affect Schedule; PCS, post-concussive symptoms; PTSD, post-traumatic stress disorder; PCA, principal component analysis; ROI, region of interest; RAVLT, Rey Auditory Verbal Learning Test; RPQ, Rivermead Post-Concussion Symptoms Questionnaire; SNP, single-nucleotide polymorphism; SHC, sparse hierarchical clustering; TBI-QOL, TBI Quality of Life; TDA, topological data analysis; UPGMC, unweighted pair-group method using centroid averages; WAIS, Wechsler Adult Intelligence Scale; ZNB, zero-inflated negative binomial.

## Inset 1: Definitions of terms.

**Subclassification**

A pathological variant of a disease or condition.

**Cluster**

A set of persons with similar traits or characteristics.

**Symptom Cluster**

A set of traits or characteristics that occur in correlation.

**Phenotype**

Any trait(s) or characteristic(s) that distinguish a specific state (e.g., a disease), or any of its subtypes.

**Phenotyping**

Processes for identifying a state or substates, distinct set of traits or characteristics.

**Supervised learning**

Algorithms for learning a function that maps input-output pairs based on a set of training examples. The function can then predict the outputs of previously unseen data.

**Unsupervised learning**

Algorithms for characterization or inference of data without labeled states. Examples include clustering and latent variable modeling for dimensionality reduction.

**Benchmarking**

The act of performing additional reference analyses, so that new systems/methods can be interpreted within a familiar context.

### Phenotyping terminology

Throughout medical literature, the term “phenotype” is interchangeably used to refer to classifications, symptoms, subtypes, clinical profiles, clusters, and observations (Inset 1). In this review, we define phenotypes to be: *any trait(s) or characteristic(s) that distinguish a specific state [TBI], or any of its subtypes*.^8^ In clinical settings, a cluster often refers to an aggregation of cases with correlated pathology (e.g., correlated through contact). Separately, “clustering” also refers to a wide category of algorithms for grouping observations according to data similarity (Inset 1); we use the latter definition in this work. Epidemiologically, a clinical phenotype may arise from the expression of one gene or, more broadly, as a correlated set of symptoms. When discussing a subtype/subclassification, we mean a pathologic variant, and when discussing its phenotype, we mean the traits that describe it. For example, a subtype may represent a pathologic variant and the trait(s) describing the variant represent its phenotype.

### Phenotyping methods

Practical approaches to phenotyping broadly divide into three areas: 1) model-based approaches such as latent class analysis (LCA), which constructs a model that assigns each case to its most representative phenotype using maximum likelihoods; 2) supervised machine-learning methods, which identify phenotypes associated with labeled data (e.g., by constructing decision tree ensembles); and 3) unsupervised machine-learning methods (typically non-parametric clustering) that search for inherent patterns and similarities between observations without the need for labels (e.g., without needing to know clinical outcomes).

Model-based approaches like LCA can uncover multiple distinct latent classes within TBI observations.^9^ In LCA, each case is assigned to any one of a set of pre-defined classes, according to its position on latent variable scale(s) derived from multi-variate data. To do this, LCA finds a set of model parameters estimated by the maximum likelihood method that optimally fits the case data. LCA is controllable and intuitive, although there is empirical evidence that rudimentary machine-learning methods can outperform LCA for medical phenotyping.^10^

Supervised learning methods, such as binomial regression, can also be used to infer predictors of TBI subtypes.^11,12^ Decision trees and random forests are also popular examples of supervised learning for TBI classification.^7^ Whereas supervised approaches predict states from a set of labeled observations, unsupervised methods—like principal component analysis (PCA) and topological data analysis^13^—allow us to probe natural patterns within data.^2^

More-recent unsupervised methods,^14^ such as hierarchical density-based scanning (HDBSCAN),^15^ can improve noise tolerance beyond traditional methods like *k*-means clustering, while also allowing for the separation of arbitrary cluster shapes (e.g., non-normal cluster presentations). Novel dimensionality reduction techniques, such as uniform manifold approximation and projection (UMAP),^16^ can complement clustering and phenotyping in high dimensions, because they can preserve the distances between high-dimensional observations in a more easily visualized, low-dimensional representation, while also maintaining a degree of global stability. However, more-sophisticated unsupervised methods, such as UMAP and HDBSCAN, have yet to be applied to TBI phenotyping.

### Review methodology

We present an expert consensus-directed, selected review of TBI classification with an emphasis on unsupervised approaches that encompass recent trends in TBI phenotyping. The database search used MEDLINE with PubMed and was limited by publication type, dates January 1, 2003 to January 1, 2020, and language (English only). Excluded publication types were comments, editorials, patient education handouts, newspaper articles, biographies, autobiographies, case reports/series, and animal studies. The literature search used the following terms: human, traumatic brain injury, phenotype, subtype, cluster, subclassification, clinical profile, and spanning. Upon expert consensus, 28 scientific publications were selected for applicability to the objective of this work, providing a state-of-the-science report of advanced approaches to TBI phenotyping (Table 1). Leveraging expert consensus, we parsed the phenotyping literature into domain subsections, presented below in order as: 1) Severity, Sources, and Populations; 2) Identified Phenotypes; 3) Severity-Based Profiles; 4) Predominantly Post-Concussion Symptom Profiles; 5) Studies Using Only Cognitive Measures; 6) Studies Using Diverse Post-TBI Measures; 7) Comorbid and Vulnerability-Based Profiles; 8) Studies Using Longitudinal, Serial Measures; 9) Studies with Postmortem Neuropathological Endpoints; and 10) TBI Phenotypes in Pediatrics.

## State of the Science

### Severity, sources, and populations

The majority of data classifying TBI focuses on post-concussion symptom predictors and profiles spanning acute through chronic injury. Acute injury was defined for this review as <2 weeks post-injury. For simplicity, chronic injury includes subacute injury and is defined as any assessment point beyond 2 weeks post-injury. Eight studies focused on the acute phase of injury, whereas 16 focused on chronic indicators. Fourteen studies assessed a range of injury severity, whereas 10 exclusively addressed mild TBI. Some studies noted that missing data precluded authors from categorizing patients based on injury severity.^17^

Population characteristics differed by age and whether participants were civilians, military, or athletes. Ages of participants in the included studies spanned exclusively from pediatric participants,^17,18^ to exclusively adults,^12,19^ to both pediatric and adult populations^3,20^; the majority of studies described TBI in adults ages ≥16 years. Civilian, active military personnel, and/or Veterans^21–24^ as well as participants with sports-specific TBI were assessed in these studies.^3,11^ Data for participants were derived from various sources, including earlier clinical trials,^4,19^ cohort studies,^12,13,23,25^ and observational health system data.^24^

### Identified phenotypes

Surprisingly, despite the diversity of populations, sources, analyses, and approaches described, the number of subclassifications were relatively consistent throughout the literature, but the interpretation of subtypes and their traits varied. Phenotypes described more than once included: 1) moderately healthy, with mild or transient symptoms, but otherwise broadly similar to controls across most measures; 2) predominantly healthy, but presumed normal, with some persistent conditions, or normal with measurable decline; 3) somatic/functional issues with moderate distress, withdrawal, pain, oculomotor, migraine, cognitive, sleep, or vestibular persistent symptoms; 4) mental health and behavioral concerns indicated by long-term depression, post-traumatic stress disorder (PTSD), migraine, substance use, and/or affective issues, often associated with higher severity; and 5) a mixed set of traits, drawn from combinations of the above, exhibiting mixed mental health, behavioral, and long-term functional concerns. Notably, within this literature base, no mechanism-driven phenotypes were reported.

### Severity-based profiles

Several studies used data from the Transforming Research Clinical Knowledge in Traumatic Brain Injury (TRACK-TBI) Longitudinal Study pilot and Citicoline Brain Injury Treatment (COBRIT) trial to identify acute injury phenotypes in adults.^4,13,19,25^ These studies adopted different analytical approaches with similar types of injury data (e.g., computed tomography [CT] scan, physiological measures, vital signs within 24 h of injury, and medical history). Nielson and colleagues used topological data analysis to examine TBI injury characteristics (acute neuroimaging results) and 6-month outcomes for PTSD diagnosis and neuropsychological testing (processing speed and verbal learning) to identify subtypes of persons with mild TBI in TRACK-TBI pilot data.^13^ Their results identified a subtype of persons with unfavorable outcomes on the Glasgow Outcomes Scale-Extended (GOSE) who also had high rates of PTSD at 6 months and who also had genetic polymorphisms associated with striatal dopamine processing, cellular stress, and DNA damage.

Si and colleagues used sparse hierarchical clustering and outcome selection on TRACK-TBI pilot data using 53 clinical variables, each collected at baseline and through emergency/hospital care. Variables tested included demographic characteristics, prior history, injury characteristics, results of lab/radiology tests in the first 24 h, loss/alteration of consciousness, or post-traumatic amnesia.^25^ They report five subtypes of mild TBI that were primarily distinguished by CT results (see Table 1). The subtypes include: 1) admission blood pressure; 2) alcohol and tobacco history; and 3) history of neurological and psychological history. Subclasses differed in outcomes after 6 months, with varying general cognitive, emotional, and physical function. For instance, one of the subtypes displayed normal CT, but significantly more tobacco use. For this subset, neurological and psychiatric history had significantly worse outcomes than the other groups on the Brief Symptom Inventory, Weschler Adult Intelligence Scale (WAIS), and the Trails Making Tests.

Two studies used COBRIT trial data to explore acute injury/treatment data collected in the first 24 h after a TBI (e.g., fluid intake, blood tests, and CT scan measures). Both studies included persons with non-penetrating TBI and positive baseline CT, but each study used a different analysis and found slightly different phenotypes. Masino et al. identified four phenotypes that were associated with 90-day outcomes on cognitive, emotional and other functional outcomes^4^, whereas Folweiler et al. used similar methods and measures but identified three phenotypes^19^. Separately, Folweiler et al. reported three phenotypes using supervised *k*-means clustering on TRACK-TBI data. Two out of the three phenotypes were similar in both the COBRIT and TRACK-TBI samples.

An analysis of acute care data collected (including imaging characteristics in the first 24 h) was undertaken in adults from the CENTER-TBI European cohort study.^22^ By applying PCA and then clustering on the reduced dimensional outputs with bootstrap resampling, they revealed four stable clusters of TBI severity (Table 1). These clusters were distinguished by 1) injury mechanism, 2) presence of extracranial injury, and 3) Glasgow Coma Scale (GCS). The results implied treating intermediate (moderate) severities as two distinct groups (upper and lower), that is, 1) mild, 2) moderate lower, 3) moderate upper, and 4) severe. Traditional severity categories are broad and encompass very different pathologies, and so research into further gradations asserted by Gravesteijn et al. could find value for assisting differential diagnosis, and/or quantifying cases where additional diagnostics are/are not necessary.

### Predominantly post-concussion symptom profiles

Several studies examined post-concussion symptom profiles using a variety of measures and analytical techniques to derive phenotypes that inform prevalence, predictors of recovery, and/or target potential treatments. Lumba-Brown and colleagues convened a multi-disciplinary expert workgroup to define common subtypes of concussion in pediatric and adult populations and identify clinical questions related to prevalence and recovery. Using these key clinical questions, the group conducted a systematic review and meta-analysis of concussion subtype prevalence.^26^ The meta-analysis of multiple measures (clinical signs, symptomology, neurocognitive and vestibulo-oculomotor testing, etc.) informed the varying acute prevalence of the following subclassifications, populated predominantly with post-concussive symptoms: 1) headache/migraine, 2) cognitive, 3) vestibular, 4) anxiety/mood, and 5) oculomotor impairments.

Maruta and colleagues examined post-concussive symptom clustering in a sample of adolescent through adult athletes participating in a variety of sports.^11^ Athletes were evaluated with a modified Rivermead Post-Concussion Symptoms Questionnaire (RPQ) at baseline and at 2 weeks after a physician diagnosed concussion. Using a binomial test to compare baseline and post-injury post-concussive symptoms, the investigators identified six phenotypes with varying degrees of overlap: 1) cognitive/fatigue, 2) vestibular, 3) oculomotor, 4) anxiety/mood, 5) migraine, and 6) sleep. Findings were similar for adult and pediatric samples.

Stein and colleagues identified persistent post-concussive symptoms (PPCS), with a focus on physical and cognitive/emotional PPCS symptom components, at 3 and 9 months after mild TBI. They used zero inflated negative binomial regression to identify risks for PPCS reporting that loss of consciousness (LOC) and post-traumatic amnesia strongly indicated PPCS compared to persons with alteration of consciousness alone. Other significant predictors included history of TBI, psychological stress before deployment, and severe deployment-related stress. Polimanti and colleagues examined polygenic risk scores for PPCS and found no significant moderate or large effect predicting PPCS,^23^ suggesting that there is no moderate-to-large degree of shared genetic components between PPCS and psychiatric or neurodegenerative disorders.

Hellstrom and colleagues used *k*-means clustering analysis and identified four mild TBI profile clusters based on indicators from the RPQ in relationship to neuroimaging results, reported levels of anxiety, depression, and global function.^27^ Classifications were differentiated by the presence of radiological findings and were 1) general low level of symptoms, 2) general high level of symptoms, 3) high level of cognitive symptoms, and 4) high level of somatic and frustration symptoms. The study found that the cognitive cluster group had lower levels of both anxiety and depression compared to the high symptom cluster group. The high-level group generally had more complaints and high absence from work than other groups, potentially attributed to pre-existing conditions.

Yeates and colleagues, on behalf of Pediatric Emergency Research Canada, used acute injury clinical data and previous history to predict post-concussive symptoms at 4 and 12 weeks after injury in a large sample (*n* = 2,323).^9^ Separate latent class models were constructed for pre-morbid and clinical groups, and data were acquired for clinical, acute, post-concussion, and pre-morbid history measures. Both the pre-morbid and clinical latent class models supported four subclassifications. Pre-morbid history variables were associated with a higher risk of symptoms at both times, including older age, female sex, and subgroup membership. Group membership in the LCA, based on just clinical presentation data, did not elicit strong independent predictors of symptoms at either testing time.

Three additional studies examined post-concussion symptom profiles. Howell and colleagues examined post-concussive symptom domains (somatic, vestibular-ocular, cognitive, sleep, and emotional) among 689 patients ages 7–30 years, in relationship to symptom duration.^28^ The study reported longer total symptom duration associated with more severe somatic and vestibular-ocular symptoms in adolescents. Kontos and colleagues conducted a retrospective, blind chart review of 236 subjects ages 11–40 years with mild TBI within 90 days of injury from two concussion clinics.^29^ Primary and secondary clinical profiles were determined based on relevant medical history, injury information, clinical interview/examination notes, reported symptoms, and cognitive and vestibular/ocular test results. Feddermann-Demont and colleagues performed a meta-analysis of data from 2416 athletes represented by 33 prospective studies to determine, among other things, what domains of clinical function should be assessed after sports-related concussion.^30^ Domains identified were cognition, dizziness and balance, emotions, headache, and vision. This study reported that clinical domains affected in the diagnosis of sport-related concussion included cognitive, vestibular, and headache/migraine. Overall, the studies examining acute post-concussion symptom profiles reveal some similarities, with symptomatic, vestibular, and cognitive symptoms all featured repeatedly, but the measurement, magnitudes, and associations between symptoms varying considerably across studies.

### Studies using only cognitive measures

DeJong and colleagues^20^ examined cognitive phenotypes in an adult cohort based on cluster analysis of California Verbal Learning Test (CVLT) scores and change in CVLT trial administration among persons with mild and moderate/severe TBI. Six subtypes were identified, each characterized by different patterns of performance: 1) performance above the mean on all components of the CVLT; 2) improvement over the course of testing; 3) low scores and demonstrated improvement; 4) low scores with decline over the trial; 5) scores starting 0.5 standard deviation above the mean on the first word recall list with significant decline over the course of testing; and 6) performance below the mean on all tasks. Subtype analyses for those with mild TBI replicated most of the findings.

Four studies used personality measures to identify post-concussion symptom profiles; two used the Minnesota Multiphasic Personality Inventory (MMPI), and two used the Personality Assessment Inventory. Those that used the MMPI each used different clustering techniques, and each found a different number of clusters. Warriner and colleagues used the original MMPI to examine an inpatient rehabilitation cohort from a Canadian rehabilitation hospital who had a single TBI (fall, assault, motor vehicle accident, bicycle accident, or pedestrian accident) and received care for injury between 1985–1996, with the mean time from injury up to 8.5 years.^31^ Using a split-sample, three-step cluster approach, they found six injury outcome subtypes: 1) normal; 2) mild somatic/pain; 3) marked disinhibition/externalizing; 4) marked internalizing; 5) externalizing; and 6) somatic.

Goldsworthy and colleagues similarly examined outcomes in a cohort of patients who received care from a Midwestern rehabilitation hospital^21^ using a two-stage hierarchical clustering approach. Analyzing data from the MMPI-2 restructured form, they identified four clusters: 1) high symptom scores across nearly all domains, indicating dissatisfaction with life, somatic complaints, significant worry, and low positive emotions; 2) discouragement and somatic/depression symptoms; 3) minor somatic complaints; and 4) no significant elevations on scales (normal). Further evaluation revealed no differences for age, race/ethnicity, education, time post-injury, or previous psychiatric history. However, there were sex differences in cluster 3 (predominantly males) and cluster 2 (predominantly females). Comparing these studies, we note a different number and character of clusters, but each study explicitly identified both a normal and a mild somatic pain cluster. However, direct comparison of the studies is difficult because of slight differences in the MMPI administered, different chronicity, populations (United States vs. Canada), and choice of clustering analysis.

Two studies using the Personality Assessment Inventory were conducted in different populations using different clustering approaches. Both included the full spectrum of TBI severity. Velikonja and colleagues used a split sample, two step clustering approach in a cohort of Canadian patients who received care in an acquired brain injury rehabilitation unit after TBI (mean time since injury 3.87 years),^32^ whereas Kennedy and colleagues used a two-step cluster approach in a post-9/11 deployed U.S. military cohort referred for neuropsychological testing after TBI an average of 30 weeks after injury.^33^ Velikonja and colleagues found seven clusters: 1) multiple high symptoms; 2) high somatic and depressive symptoms; 3) high depression; 4) normal; 5) high substance use disorder with antisocial personality features; 6) presumed normal with possible minimalization; and 7) multiple high symptoms with borderline personality features.^32^ Kennedy and colleagues found four clusters: 1) high distress; 2) somatic; 3) moderate distress; and 4) no distress. Each study found clusters consistent with high distress and no distress/normal.^33^ Both studies also found that high distress clusters were significantly older. The researchers subsequently identified four clusters in a related work,^34^ leveraging the Neurobehavioral Symptom Inventory and PTSD Checklist-Civilian Version (PCL-C). They identified favorable outcomes, PTSD, cognitive symptoms, and mixed presentation as phenotypes.

### Studies using diverse post traumatic brain injury measures

Three studies used measures of cognitive, emotional, self-perception, and physical symptoms, in post-moderate/severe TBI. Zimmermann and colleagues performed a cluster analysis of higher-level cognition and executive function of 84 adult outpatients with mild and moderate/severe TBI.^35^ They identified three subtypes using hierarchical clustering on acute patient *z*-scores on a battery of tests. The clusters identified were: 1) inhibition, flexibility, and focused attention; 2) inhibition, flexibility, working memory, and focused attention; and 3) no expressive executive deficits. Sherer and colleagues examined outcomes of a cohort of persons who received TBI rehabilitation care in different regions and who were on average 6.3 years post-injury.^36^ They used *k*-means clustering and split half sampling, revealing five clusters: 1) normal cognition, good environmental support, and negative signs and symptoms; 2) normal cognition, intermediate environmental support, and higher emotional distress; 3) intermediate cognition, high emotional/behavioral symptoms; 4) low cognition, intermediate environmental support, lower emotional/behavioral symptoms; and 5) low cognition, high emotional and behavioral symptoms.

Juengst and colleagues interrogated results of neuropsychological testing from the PHQ-9 and other self-report measures using two-step clustering with log-linear differences and *k*-means clustering for early (<6 months after injury) and late (>6 months after injury; mean 7.2 years) recovery cohorts^37^ and split half analysis. The analyses revealed two clusters for early recovery (depressive/behavioral symptoms; no depressive/behavioral symptoms) and four clusters for the late recovery cohort: 1) poor emotional/behavioral with no cognitive impairment; 2) good emotional, average behavioral, and mild deficit to normal cognition; 3) high positive and negative affect, poor behavior, and mild deficit to normal cognition; and 4) good emotion, moderately poor behavior, and severe cognitive impairment. Despite the similarity in time post-injury and TBI characteristics between the Juengst and colleagues late recovery and Sherer and colleagues cohorts, many of the phenotype descriptions vary. One possible reason for this is the use of different measures in the cluster analysis. It is also possible that the addition of environmental support variables and participation measures included by Sherer and colleagues influenced the dispersion of mild and intermediate cognitive deficits given that there appear to be more clear demarcations of mild and moderate cognitive impairment in that study. Comparison of these three studies with standardized tools truncating to shared measures could potentially lead to synchronous results.

### Comorbid and vulnerability-based profiles

Kucukboyacl and colleagues conducted two-step cluster analysis of history of vulnerability before TBI and compared inpatient rehabilitation outcomes by cluster.^38^ Four clusters were revealed: 1) substance use and psychiatric history; 2) racial/ethnic minority and some language barrier; 3) substance use, incarceration, or homelessness history with high language barrier; and 4) elderly with complex medical comorbidity. The racial/ethnic minority group had the shortest hospitalization and showed low improvements on Functional Independence Measure scores. This study highlights how phenotypes can evolve over time and indicates that baseline characteristics related to vulnerability may be critical in understanding divergence of phenotypes.

### Studies using longitudinal, serial measures

Pugh et al. identified longitudinal comorbidity phenotypes in post-9/11 Veterans with mild TBI who sought regular Veterans Affairs (VA) care.^24^ Using health system data, they identified 22 conditions common after mild TBI including neurosensory, mental health, neurodegenerative, pain, and sleep conditions. The study conducted latent class analyses in order to identify patterns of comorbidity in the first five years after entering VA care. These data reported five comorbidity subtypes by comparing data at years 1 and 5: 1) moderately healthy, with low probabilities of any PCS, pain, or mental health-related diagnoses; 2) moderately healthy with decline, exhibiting low probabilities of any relevant diagnoses for year 1 and high probabilities of mental-health–, pain-, and PCS-related diagnoses at year 5; 3) mental health (high probabilities of PTSD, substance use disorder, and depression); 4) polytrauma, with high probabilities of mental health–, pain-, and PCS-related diagnoses; and 5) polytrauma with improvement, with initially high mental health and pain, but showing significantly lower probabilities at year 5. Suicidal ideation and attempt were phenotypes of the mental health subtype and were not phenotypes of the polytrauma with improvement subtype.

### Studies with post-mortem neuropathological end-points

There is increasing awareness of the value of studying brain tissue collected from persons with TBI from well-characterized clinical cohorts to permit investigation into the neuropathological correlates of distinct TBI exposure patterns and clinical presentations. The Late Effects of TBI (LETBI) study is one such multi-center, prospective, longitudinal study designed to characterize *in vivo* phenotypes of chronic TBI and their underlying neuropathology.^39^ Early investigations of brain tissue studied using LETBI multi-modal autopsy methods (high respolution *ex vivo* neuroimaging, image-guided tissue sectioning for standard and targeted neuropathological examination, and structured family interview complemented with medical record abstraction) reveal the complex coexistence of multiple pathological proceses associated with diverse injury exposure histories and clinical presentations.^40,41^ This work underscores the importance of collecting accurate information about lifetime exposure to TBI of diverse etiolgoies, in addition to deep clinical phenotyping to investigate alongside post-mortem pathological findings. Challenges inherent to studing the pathology of moderate-severe TBI with imaging biomarkers have necessitated the development of novel methods to correct for large lesions,^42^ to facilitate image processing and allow inclusion of these valuable cases with more severe TBI in multi-modal phenotyping studies.

As we await the maturation of prospective studies, structured post-mortem family interview methods have been developed and used to examine the associations of repetitive TBI exposure symptom profiles with chronic traumatic encephalopathy neuropathological lesions,^43^ and inclusion of detailed lifetime TBI exposure questionnaires in ongoing longitudinal studies of aging have permitted similarly impactful investigation into the neuropathology underlying clinical symptoms of dementia and other neurodegenerative diseases.^44^ Ongoing investment into studies investigating the pathophysiological underpinnings of distint TBI phenotypes will be critical for identifying novel targets for intervention and aligning new treatments with the patients who can most benefit from them.

### Traumatic brain injury phenotypes in pediatrics

Several studies that explored pediatric cohorts have already been described in other contexts.^3,9,11^ In other work, different phenotyping methods have reported distinct profiles from normal to pervasive emotional difficulties after pediatric TBI.^17,18^ Four cognitive subtypes of psychometric measures (including Personality Inventory for Children Revised, the Behavior Assessment System for Children-Second Edition [BASC-2], and the Weschler Intelligence Scale for Children-Third Edition) were identified. Importantly, neither the work by Ensign or Hayman found any relation between injury severity and subtype membership.

Another study examined the results of the CVLT using a two-stage cluster analysis method that used Ward's minimum variance agglomerative clustering technique and *k*-means iterative partitioning.^45^ The analysis revealed four clusters with distinct phenotypes, which differed on *z*-scores of attention span, learning efficiency, long delay free recall, and number of false positives. They were: 1) low scores on list A, 2) low scores on list A and few false positives, 3) similar scores across all measures, and 4) high scores on list A, long-delay free recall, and the lowest false positive scores. Below-average groups were more likely to have experienced extended coma.

## PhenoBench: Benchmarks for Phenotyping

**FIG. 2.**
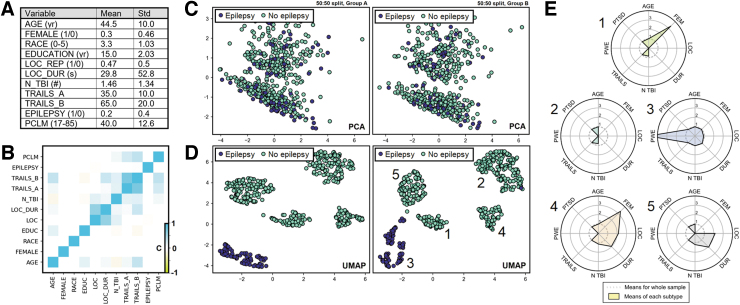
PhenoBench data generation and example outputs from a synthetic post-traumatic epilepsy (PTE) data set. (**A**) Inset table of means and standard deviations for the synthetic data set (*N* = 1000). Explanation of the variables are provided in the documentation. (**B**) Heatmap of the correlation matrix for the 11 synthetic variables. (**C**) PCA two-principal component reduction of the pseudo data set, shown for two random 50/50 split samples of the data (left, right). Trends in the global structure are similar across subsamples, indicating good group stability, but the PTE group differences are not captured by PCA. (**D**) Like (C), but with a UMAP reduction of the synthetic data set down to two dimensions. Trends in the global structure are broadly similar, and the PTE phenotype is distinct. (**E**) Phenotypes are shown in a radial plot broken out across the five clusters found by UMAP embedding in (D). PCA, principal component analysis; UMAP, uniform manifold approximation and projection. Color image is available online.

Our review reports strong analytical diversity throughout the field, which presents unique challenges for the comparison and reproduction of phenotypes across reports. Uncovering distinct trajectories and phenotypes can assist clinicians, but the current literature sampled in this review does not provide a clear path to operationalized phenotypes for clinical practice. In order to address this limitation, we provide a free, open access repository called PhenoBench,^46^ that uses state-of-the-art unsupervised machine-learning tools to uncover distinct phenotypes within raw data. These tools are intended for researchers to analyze their data offline to identify phenotypes and subtype groups using a standardized approach. The purpose of the repository is not to require any particular analysis pipeline, but, instead, to offer a standard set of analysis methods that can be run in parallel to a preferred analysis (Fig. 2). The primary output of the repository is phenotype labels for each observation and statistical measures of ranked trait importance of subtypes, which can be assessed across studies or data sets. The repository contains 1) a synthetic data-generating tool (Fig. 2A,B) based on TBI covariates; 2) a set of dimensionality reduction and clustering pipelines (called P_A_ and P_B_); and 3) additional tools for statistical and graphical analysis.

### Summary of features

Benchmark 1 (P_A_) combines PCA for dimensionality reduction with *k*-means clustering, which requires the user to specify a putative number of separable groups. Benchmark P_B_ implements the dimensionality reduction algorithm UMAP^16^ down to two dimensions and unsupervised labeling with HDBSCAN.^15^ P_B_ does not require the user to provide an estimated number of groupings and can tolerate non-normally distributed data. The two benchmarks (P_A_, P_B_) are purposely chosen to represent orthogonal perspectives (Fig. 2C,D).

The primary outputs are tables and radial plots of the mean/median traits within each cluster/subclass identified (Fig. 2E). After package installation, both benchmarks are performed by running a single Python script. To compare across data sets, summary statistics can be produced for each data set and compared, or if both raw data sets are available to a single user, they can be aggregated for mega analysis.

### Statistical evaluation

Figure 2 shows example output run on the included synthetic data set. PhenoBench implements a generic mean silhouette score for assessing the separability of output clusters. To assess the stability of clusters, PhenoBench can also perform split sampling (see Fig. 2C,D). To ascertain what traits are associated with each group, we derive the mean value of each trait broken out by cluster as a ratio of the total sample mean (see Fig. 2E). The extent to which a phenotype is truly distinct depends on how uniquely a symptom profile can be attributed to a particular group, and so the likelihood of the observed means per group is provided as a measure of statistical strength of phenotypes.

## Conclusion

The heterogeneity and complexity of TBI recovery and prognosis extends beyond conventional severity scales. Here, we present a literature review of recent trends in TBI phenotyping and find that four to five phenotypes are often indicated, irrespective of field or population, although there are significant variations in methodology and inclusion criteria. We detail five phenotypes that arise more than once across studies, but most phenotypes described are inconsistent across reports. In the absence of standardization, it is unclear how to consolidate these disparate observations. To address this, we provide a freely available phenotyping pipeline (PhenoBench), to encourage standardization, benchmarking, and statistical validation of TBI phenotyping. The next steps include collaboration to uncover reproducible TBI phenotypes across sites, studies, and populations. For clinical relevance, we summarize our findings with five takeaways and four trackable recommendations that include specific action items (Table 2):

**Table 2.** Actionable Research Recommendations for TBI Phenotyping

Recommendation	Action
**1.** Establish patient phenotypes by unifying and harmonizing data to understand differences and similarities across populations	• TBI research, diagnosis, management, recovery, and prognosis make use of a broad and often disparate spectrum of measures. Where appropriate, incorporating multiple data types per study can advance our understanding of phenotypes by promoting interdisciplinary perspectives.
**2.** Develop, validate, and standardize tools and assessments	• When designing new studies, consider analysis pipelines and tools used in earlier phenotyping reports. • Incorporate the use of common data elements to improve collaboration and synthesis of findings. • Renew efforts toward data sharing, consistency, and collaboration to provide common reference points.
**3.** Develop registries or repositories, democratize data, and prioritize privacy	• Use public data sets to explore and validate methods and outcomes at scale. • Adopt policies for tool sharing, which enables valuable comparisons and validation of phenotypes across studies and data sets. • Adopt policies for knowledge sharing of negative or contradictory results, which will allow the community to identify contexts that confound conventional understanding.
**4.** Identify and integrate constellations of phenotype data from different modalities	• Efforts to identify phenotypes within and across populations should be driven by the need to target the right participants into the right TBI clinical trials and accelerate treatments into practice.

TBI, traumatic brain injury.

1. TBI is a heterogeneous injury with varying symptoms, impairments, and recovery patterns across similar TBI severity levels.
2. There is inconsistency in existing phenotyping terminology, methods, measures, and populations; this results in thematically distinct TBI subclassifications lacking consistent clinical relevance.
3. Establishing common methods, measures, and means for tool sharing will help clarify our understanding of TBI phenotypes.
4. Recent large, deep phenotyped TBI cohort studies offer an opportunity to develop a new taxonomy based on mechanism.
5. Establishing shared data repositories allows for phenotypic enrichment and can drive precision medicine models.
